# Genome-Wide Characterization and Expression Profiling of the Chitinase Gene Family in Radish (*Raphanus sativus* L.) Under Clubroot Stress

**DOI:** 10.3390/ijms27156765

**Published:** 2026-07-28

**Authors:** Zhijie Liu, Tianhua Hu, Minyan Mai, Wuhong Wang, Qingzhen Wei, Haijiao Hu, Yaqin Yan, Chonglai Bao, Yaowei Zhang, Jinglei Wang

**Affiliations:** 1 College of Horticulture, Northeast Agricultural University, Harbin 150030, China; liuzhijie2025@126.com; 2 Vegetable Research Institute, Zhejiang Academy of Agricultural Sciences, Hangzhou 310021, China; huth@zaas.ac.cn (T.H.); maiminyan2003@outlook.com (M.M.); wangwh@zaas.ac.cn (W.W.); weiqz@zaas.ac.cn (Q.W.); huhj@zaas.ac.cn (H.H.); yanyq@zaas.ac.cn (Y.Y.); baocl@zaas.ac.cn (C.B.)

**Keywords:** *Raphanus sativus*, chitinase, gene family, clubroot resistance, *Plasmodiophora brassicae*

## Abstract

Clubroot, caused by the obligate biotrophic protist *Plasmodiophora brassicae* (*P. brassicae*), is a destructive soil-borne disease that severely threatens the production of radish (*Raphanus sativus* L.). Although chitinases are known to execute critical defense functions by degrading pathogen chitin, a comprehensive genome-wide characterization of the radish chitinase (*RsChi*) gene family and its specific role in clubroot resistance remains lacking. Here, we systematically identified 24 *RsChi* genes in the radish genome, characterizing their chromosomal distribution, structural organization, and promoter regulatory networks. These genes are unevenly distributed across seven chromosomes and cluster into four subfamilies, with tandem duplication driving family expansion, particularly on Chromosome 3. Promoter analysis revealed a significant enrichment of jasmonic acid- and abscisic acid-responsive cis-elements, implicating *RsChi* genes in hormone-mediated defense signaling. Using qRT-PCR to profile expression dynamics during *P. brassicae* infection across contrasting radish lines, we identified strong genotype- and stage-specific transcriptional responses. Notably, *TRs0x1c000780* remained transcriptionally silent prior to infection but was specifically induced over 10-fold in the resistant line at 28 days post-inoculation. This infection-triggered induction positions *TRs0x1c000780* as a promising candidate defense gene. Together, these findings provide structural and functional insights into the *RsChi* family and highlight candidate targets for breeding clubroot-resistant radish cultivars.

## 1. Introduction

Radish (*Raphanus sativus* L., 2n = 18) is an economically vital root vegetable crop, cultivated globally, exhibiting extensive phenotypic diversity in taproot morphology, pigmentation, and nutritional quality [1,2]. However, radish yield and quality are severely threatened by clubroot, a devastating soil-borne disease caused by the obligate biotrophic protist *Plasmodiophora brassicae* [3,4]. Because *P. brassicae* resting spores can persist in soil for decades, traditional chemical control and agronomic practices remain insufficient for sustainable disease management [5,6].

Plants employ a two-tiered innate immune system to counteract microbial pathogens, comprising pattern-triggered immunity (PTI) and effector-triggered immunity (ETI) [7,8]. The accumulation of pathogenesis-related (PR) proteins marks a pivotal step in host defense deployment [9,10]. Among them, PR-3 family chitinases (EC 3.2.1.14) serve as essential defense enzymes [11,12]. Chitinases catalyze the endo-hydrolytic cleavage of beta-1,4-glycosidic linkages in chitin, releasing N-acetylglucosamine oligomers [13]. Although chitin is absent in plants [14], it represents a major structural constituent of fungal cell walls and *P. brassicae* resting spore walls [15]. Based on sequence homology and domain architecture, plant chitinases belong to glycosyl hydrolase families GH18 and GH19 [16], which are further organized into five classical subfamilies (Class I to V) [17,18].

Chitin metabolism represents a conserved target for biological control across kingdoms [19]. Alongside chitinases, chitin deacetylases (CDAs) alter chitin properties by removing N-acetyl groups in fungi and insects [20,21,22]. In plant–pathogen interactions, chitinase-mediated cleavage of cell wall chitin directly restricts pathogen invasion while generating elicitor-active chitin oligosaccharides (CTOS) [23]. These CTOS molecules act as potent pathogen-associated molecular patterns (PAMPs), binding host cell-surface pattern recognition receptors (PRRs) to trigger intracellular defense signaling and systemic acquired resistance [24].

High-throughput sequencing has enabled genome-wide characterization of chitinase gene families in several Brassicaceae species, including *Brassica oleracea*, *Brassica rapa*, and *Brassica juncea*, revealing shared structural motifs and stress-responsive patterns [12,25]. Transcriptomic analyses show that specific chitinase subfamilies are dramatically recruited during fungal and protist infections [25]. Phytohormone signaling networks, particularly jasmonic acid (JA), ethylene (ET), and salicylic acid (SA) pathways, tightly orchestrate host resistance against *Plasmodiophora brassicae* [26], whereas pathogen-driven modulation of auxin (AUX) homeostasis promotes root gall formation in susceptible hosts [4,27]. However, the evolutionary dynamics of the chitinase (*RsChi*) gene family in radish and its spatiotemporal expression profiles under clubroot challenge remain to be elucidated.

To address this knowledge gap, we performed a comprehensive genome-wide identification of the *RsChi* gene family using a high-quality *Raphanus sativus* reference genome. We systematically characterized their physicochemical features, chromosomal organization, phylogenetic relationships, conserved motifs, and promoter cis-regulatory networks. Furthermore, using quantitative real-time PCR (qRT-PCR), we profiled the expression dynamics of *RsChi* genes across key infection stages in contrasting resistant and susceptible radish inbred lines. These analyses identified a prominent apoplastic candidate defense gene, *TRs0x1c000780*, which exhibited strong late-stage induction in the resistant host. Overall, this study provides structural and functional insights into the *RsChi* family, establishing a valuable resource for molecular breeding of clubroot-resistant radish cultivars.

## 2. Results

### 2.1. Identification and Physicochemical Properties of RsChi Genes

A total of 24 putative chitinase genes were identified in the *Raphanus sativus* genome (Assembly IVFCAAS_Rs00_1.1, Accession: GCA_019703475.1) through HMM and BLASTP (version 2.15.0) searches. All candidates were further verified by confirming the presence of the conserved GH18 or GH19 domains. These genes were listed with their original accession numbers as retrieved from the genome of *R. sativus* cv. Xin-li-mei, which was obtained from the Raphanus genome dataset (Table 1).

The physicochemical properties of the chitinase proteins varied significantly. The *RsChi* proteins ranged in length from 158 (*TRs0x3c013268*) to 475 (*TRs0x6c030662*) amino acids, with molecular weights ranging from 16.9 to 51.5 kDa. Theoretical isoelectric points (pI) ranged from 4.52 to 9.18, with 11 acidic proteins (pI < 7) and 13 basic proteins (pI > 7).

### 2.2. Chromosomal Distribution and Gene Duplication of RsChi Genes

**Figure 1:**
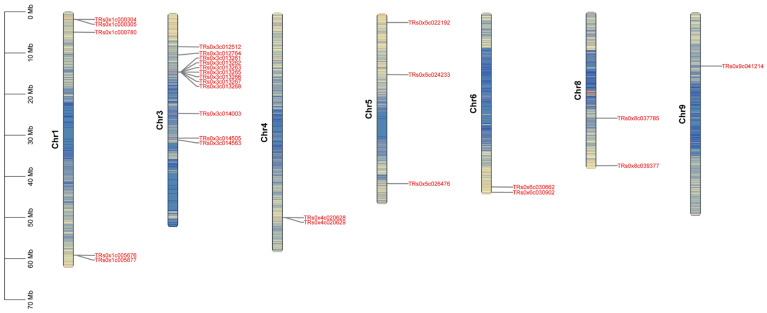
Chromosomal distribution of *RsChi* genes across *Raphanus sativus* chromosomes. The chromosome numbers are indicated at the top of each vertical bar, and the physical positions (Mb) are shown on the left scale. The gene names are shown on the right side of the chromosomes.

Physical mapping located the 24 *RsChi* genes onto the *Raphanus sativus* chromosomes. As shown in Figure 1, these genes are unevenly distributed across seven chromosomes (Chr1, 3, 4, 5, 6, 8, and 9). We identified no *RsChi* genes on Chr2 or Chr7. Chr3 contains the largest group with 10 members, representing 41.7% of the family, followed by Chr1 with five genes. Chr5 contains three genes, Chr4 and Chr6 contain two genes each, and Chr8 and Chr9 harbor one gene each.

### 2.3. Phylogenetic Analysis and Subfamily Classification

**Figure 2:**
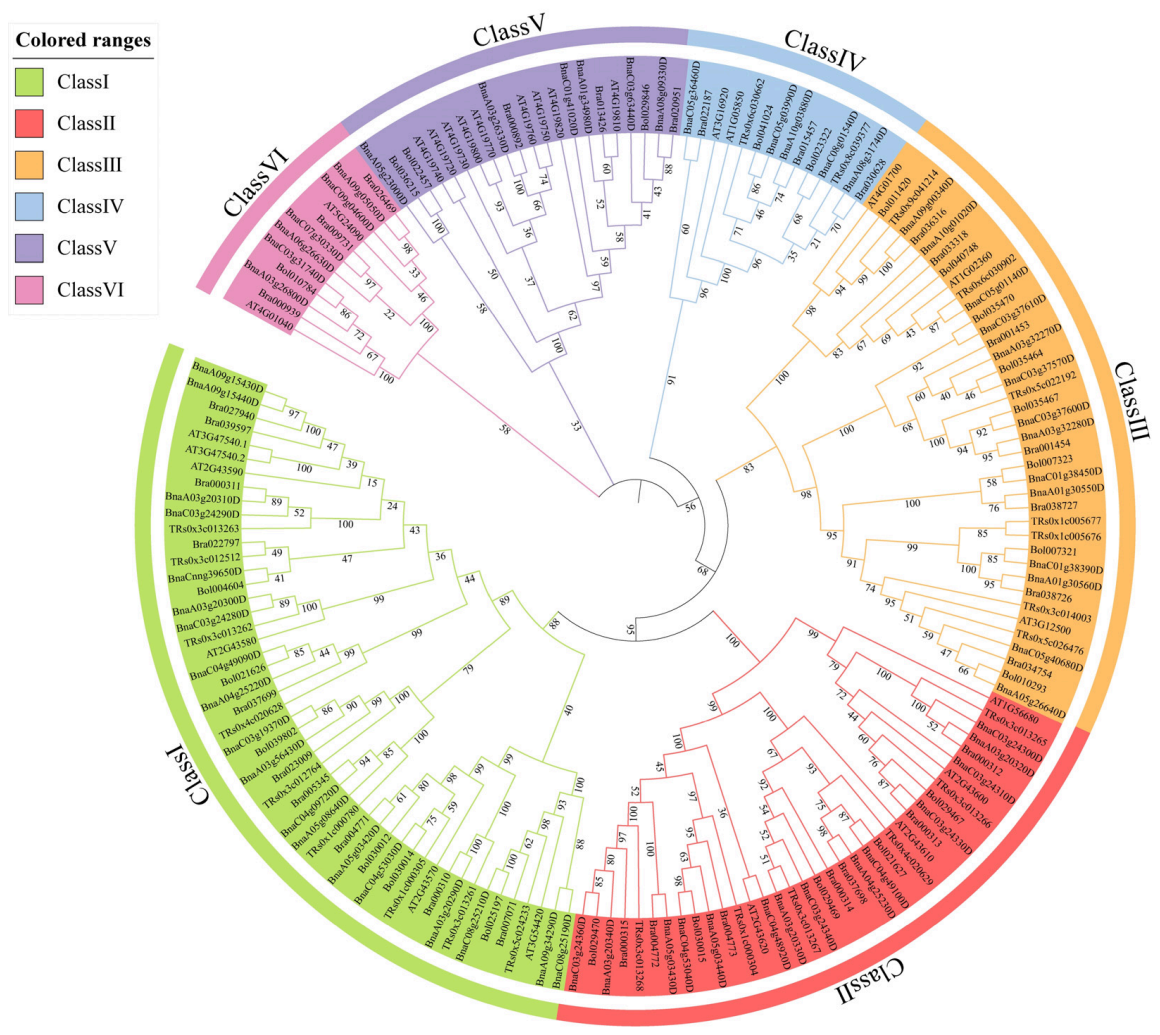
Unrooted phylogenetic tree of chitinase proteins from *Raphanus sativus*, *Arabidopsis thaliana*, *Brassica napus*, *Brassica rapa*, and *Brassica oleracea*. The tree was constructed using MEGA 11 with the Neighbor-Joining (NJ) method and 1000 bootstrap replicates. The different subfamilies (Class I to VI) are marked with distinct colors.

Phylogenetic reconstruction yielded an unrooted phylogenetic tree using 173 full-length chitinase sequences from five Brassicaceae species. This dataset included sequences from radish, Arabidopsis, and three Brassica species. Based on this phylogeny, we divided the chitinases into six classes (Class I to VI). The 24 radish chitinase genes clustered into four of these classes (Figure 2).

We analyzed gene duplication events and identified clustering on Chr3. A high-density cluster of seven tandemly arrayed genes (*TRs0x3c013261* to *TRs0x3c013268*) resides on Chr3. Additional tandem duplication pairs occur on Chr1 (*TRs0x1c000304/TRs0x1c000305* and *TRs0x1c005676/TRs0x1c005677*) and Chr4 (*TRs0x4c020628/TRs0x4c020629*).

### 2.4. Gene Structure, Conserved Motifs, and Domain Architectures

**Figure 3:**
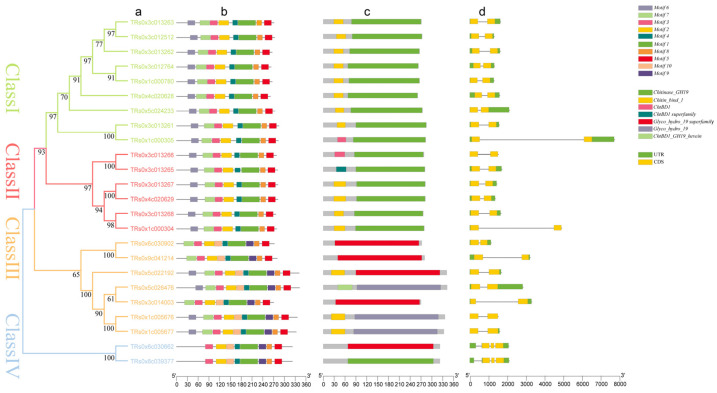
Phylogenetic relationships, conserved protein motifs, domain architectures, and ex-on-intron structures of the *RsChi* gene family. (**a**) Unrooted phylogenetic tree of 24 *RsChi* proteins; (**b**) Distribution of 10 conserved motifs identified by the MEME suite; (**c**) Conserved domain architectures analyzed by the NCBI Conserved Domain Database; (**d**) Exon-intron structures of *RsChi* genes (exons are represented by green boxes, introns by black lines).

The 24 *RsChi* genes are unevenly distributed across these subfamilies. Class I is the largest group, containing 9 members, including 3 tandemly duplicated genes on Chr3 (*TRs0x3c013261*, *TRs0x3c013262*, and *TRs0x3c013263*). Class III is the second largest subfamily, containing 7 members. Class II contains 6 members, including the remaining tandemly duplicated genes on Chr3 (*TRs0x3c013265*, *TRs0x3c013266*, *TRs0x3c013267*, and *TRs0x3c013268*). Class IV is the smallest subfamily, containing only 2 radish genes (*TRs0x6c030662* and *TRs0x8c039377*). We identified no radish chitinase genes in Class V or Class VI (Figure 3).

Domain architecture analysis revealed that all 24 *RsChi* members contain characteristic glycosyl hydrolase domains, such as Glyco_hydro_19. Several Class I and Class IV members also contain N-terminal or internal chitin-binding domains. These auxiliary modules include Chitin_bind_1 and ChtBD1 domain families (Figure 3).

We identified 10 distinct motifs (Motifs 1 to 10) using the MEME suite. Class I and Class II members share a conserved motif combination flanking the catalytic domain. In contrast, Class III members contain specific motifs (such as Motif 9) while lacking some motifs found in Class I and Class II (Figure 3).

Gene structure analysis revealed variation in gene structures across the family. Most *RsChi* genes contain two or three exons in their coding sequences. A few members contain only one exon, while others have up to four exons.

**Figure 4:**
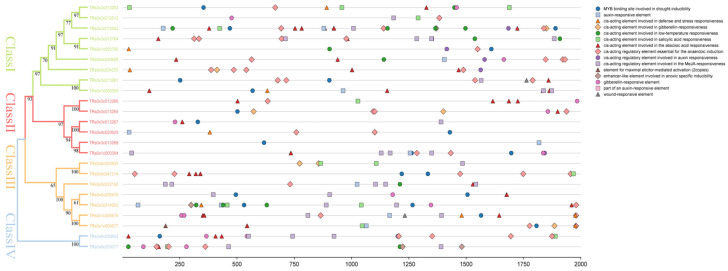
Predicted stress- and hormone-responsive cis-acting regulatory elements in the promoter regions of *RsChi* genes. The promoter regions (2000 bp upstream of the start codon) were analyzed using the PlantCARE database. Different colored boxes represent distinct regulatory elements, and their positions are mapped along the promoter sequences.

In silico promoter profiling identified potential cis-acting regulatory elements in the promoter regions of *RsChi* genes. Using the PlantCARE database, we identified 15 stress- and hormone-responsive elements (Figure 4).

We categorized these elements into hormone-responsive and stress-responsive groups. The hormone-responsive elements include ABRE, CGTCA-motif, GARE-motif, AuxRR-core, TCA-element, Auxin-responsive element, P-box, and TGA-element. The stress-responsive elements include ARE, MBS, TC-rich repeats, LTR, GC-motif, WUN-motif, and elicitor-mediated activation elements. All 24 *RsChi* genes contain between 2 and 26 of these elements. ABRE and ARE motifs are the most abundant, occurring in 19 *RsChi* genes. We detected the jasmonate-responsive CGTCA-motif in 14 *RsChi* genes. Additionally, 16 genes contain drought-inducible MBS elements, 10 contain salicylic acid-responsive TCA-elements, and 8 contain defense-responsive TC-rich repeats.

### 2.5. Expression Profiling of RsChi Genes Under Clubroot Stress

**Figure 5:**
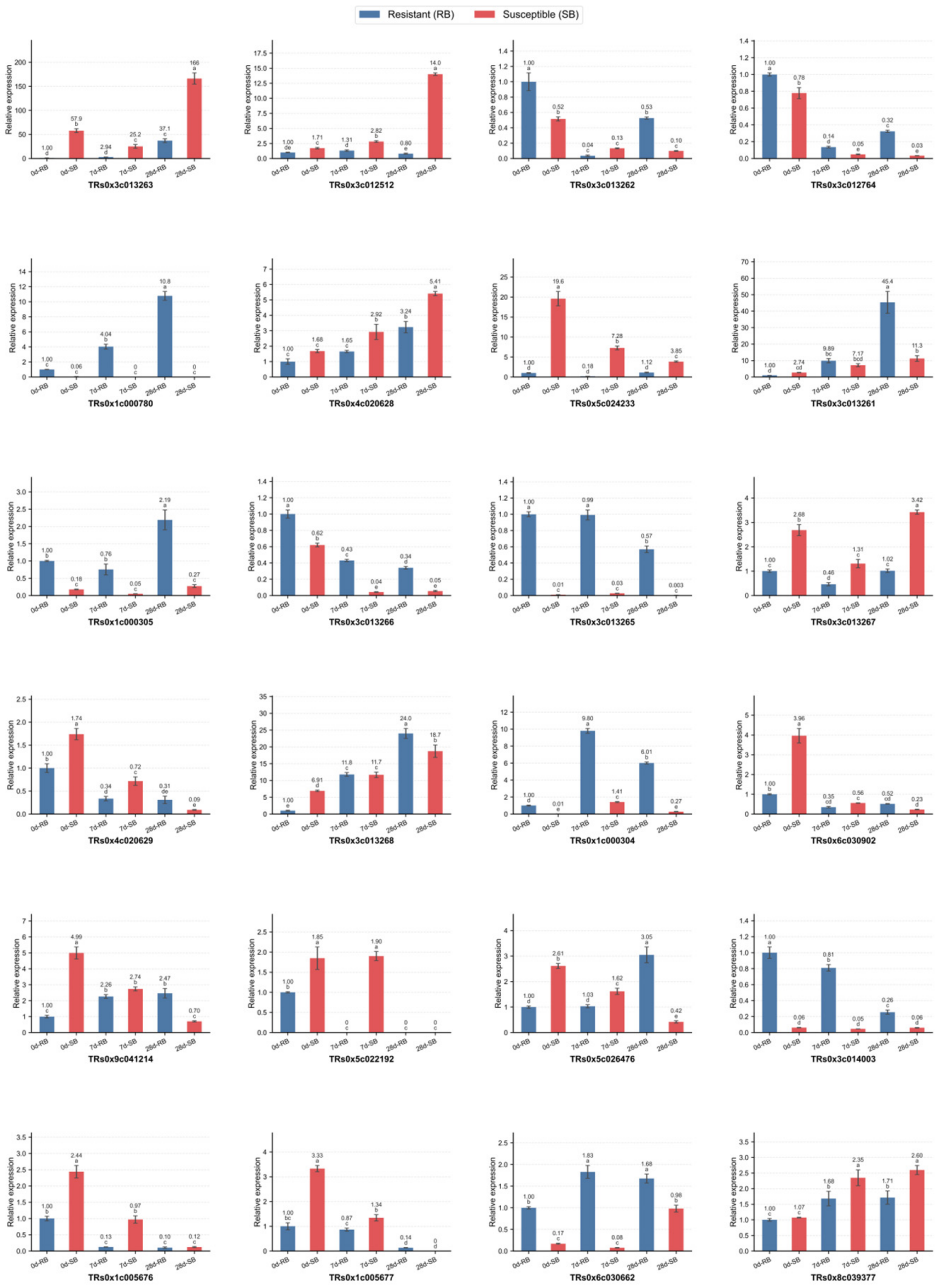
Expression profiling of *RsChi* genes in roots of resistant (RB) and susceptible (SB) radish lines at 0, 7, and 28 days after inoculation (DAI) with *Plasmodiophora brassicae*. Transcript levels were determined by qRT-PCR using *RsGAPDH* as the internal reference. The error bars represent the standard error of three biological replicates. Different lowercase letters (a, b, c, d, e) indicate statistically significant differences between different treatment groups and time points determined by two-way ANOVA followed by Tukey’s HSD test (*p* < 0.05). Shared letters indicate no significant difference (*p* ≥ 0.05).

Quantitative real-time PCR (qRT-PCR) profiling revealed expression dynamics of *RsChi* genes during infection. We measured transcript levels in resistant and susceptible roots at 0, 7, and 28 days after inoculation (Figure 5).

The qRT-PCR analysis showed genotype- and stage-specific expression profiles. *TRs0x5c026476* maintained high baseline expression across all stages and lines, suggesting a role in constitutive defense. Notably, *TRs0x1c000780* exhibited undetectable expression prior to infection (0 DAI) and at 7 DAI, but was induced over 10-fold in the resistant line at 28 DAI while remaining silenced in the susceptible line.

### 2.6. Subcellular Localization of TRs0x1c000780

**Figure 6:**
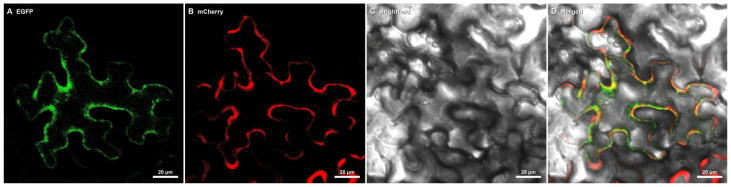
Subcellular localization of *TRs0x1c000780*-GFP fusion protein co-expressed with *Arabidopsis* marker *AT2G14610.1*-mCherry in *Nicotiana benthamiana* leaf epidermal cells. The 35S::*TRs0x1c000780*-GFP construct (green) and 35S::*AT2G14610.1*-mCherry construct (red) were transiently co-expressed via *Agrobacterium*-mediated infiltration. Confocal laser scanning microscopy was used to capture EGFP (green), mCherry (red), Brightfield, and Merged channels. Scale bars = 20 μm.

We constructed a 35S::*TRs0x1c000780*-GFP fusion vector and co-expressed it with the apoplastic marker 35S::*AT2G14610.1*-mCherry in *Nicotiana benthamiana* leaf epidermal cells. Laser scanning confocal microscopy demonstrated complete overlap between GFP fluorescence and mCherry signal (Figure 6), confirming that *TRs0x1c000780* is specifically targeted to the apoplast/extracellular space.

## 3. Discussion

### 3.1. Evolutionary Dynamics and Lineage-Specific Expansion of the RsChi Gene Family

Pathogenesis-related (PR) proteins, particularly chitinases of the PR-3 family, execute plant innate immunity against invading pathogens [9,10]. Because chitinases degrade pathogen cell walls, these gene families face constant evolutionary pressure [11,28]. We identified 24 *RsChi* genes using a high-fidelity *Raphanus* reference genome [2]. Comparative genomic strategies provide key insights into gene family evolution across Brassicaceae lineages [29,30]. The *RsChi* family is smaller than families in Arabidopsis and other Brassicaceae species. For example, *Brassica rapa* and *Brassica juncea* contain 33 and 26 members, respectively [12,25]. This genomic contraction may reflect lineage-specific gene loss during the post-polyploid evolutionary process in radish [31,32,33]. Despite this contraction, we observed a dense tandem duplication cluster on Chromosome 3. This cluster contains genes from Class I and Class II. Tandem arrays predominantly drive the expansion and functional diversification of plant chitinases [34,35]. The retention of these duplicate classes suggests that radish maintains a concentrated chitinolytic system to resist environmental stress. This pattern of tandem expansion on specific chromosomes is consistent with findings in *Brassica rapa*, where tandem duplication events on chromosomes A03 and A10 significantly expanded the chitinase family [25]. Furthermore, similar lineage-specific expansion via tandem duplication was observed in other Brassicaceae polyploids, such as *Brassica juncea* and *Camelina sativa*, where tandem clusters represent major expansion events for chitinase subfamilies under pathogen stress [12]. These comparative genomic features corroborate that while genomic contraction occurred globally during diploidization, local tandem duplications were selectively retained in Brassicaceae genomes to preserve essential pathogen-responsive genes.

### 3.2. Structural Conservation and Complex Hormonal Regulatory Networks

Domain architectures provide the structural foundation for chitinase functions. All 24 *RsChi* members retain the conserved GH19 catalytic domain [16]. This domain enables the cleavage of internal beta-1,4-glycosidic linkages in chitin [13]. Several *RsChi* members also contain auxiliary chitin-binding modules, such as the ChtBD1 domain. Studies in insect and fungal systems suggest that these binding domains enhance affinity to insoluble chitin matrices [20,35]. This enhanced affinity may boost overall antifungal efficacy during pathogen infection [18].

Complex phytohormone networks tightly orchestrate the spatiotemporal activation of these defense weapons [7]. Jasmonic acid (JA), ethylene, and abscisic acid (ABA) signaling pathways mediate clubroot resistance in Brassica hosts [26,36]. Conversely, *Plasmodiophora brassicae* alters auxin homeostasis to promote root gall development in susceptible plants [4,27]. Salicylic acid also participates in basal defense, though its role remains less clear [37,38]. Our promoter predictions match these models, showing enrichment of W-box motifs (WRKY-binding sites), CGTCA-motifs (jasmonate-responsive), and ethylene-responsive elements (ERE) in *RsChi* genes. This enrichment suggests that JA and ABA signaling pathways directly regulate *RsChi* expression, enabling radishes to dynamically reprogram their immunity against diverse stresses [39,40]. The predominance of JA- and ABA-responsive cis-elements in RsChi promoters directly correlates with expression dynamics observed under stress. In *Brassica rapa*, chitinase genes were shown to be transcriptionally responsive to exogenous methyl jasmonate (MeJA), abscisic acid (ABA), and salicylic acid (SA) treatments [41]. Furthermore, our results corroborate the classic models of phytohormone-mediated defense in Arabidopsis, where JA-dependent pathways play a central role in activating chitinase expression to restrict biotrophic pathogens [26]. This pattern conservation of promoter architecture across Raphanus, Brassica, and Arabidopsis highlights a shared evolutionary mechanism in Brassicaceae for coordinating chitinase expression through stress-responsive hormonal networks.

### 3.3. StageSpecific Transcriptional Reprogramming Highlights the Role of TRs0x1c000780

Clubroot is highly destructive, because *Plasmodiophora brassicae* spores persist in soil for decades [3,6]. The interaction between hosts and *P. brassicae* involves PTI and ETI pathways [24,42]. Significant transcriptional reprogramming occurs during secondary infection, which is largely driven by ETI [43,44]. Our qRT-PCR profiling revealed genotype- and stage-specific expression of *RsChi* genes. In susceptible roots, we observed pathogen-triggered suppression of highly expressed genes, such as *TRs0x5c026476*. This suppression may reflect the action of *P. brassicae* effectors that hijack host transcription to promote gall formation [4,42].

In contrast, the resistant line mounted a robust late-stage defense response. Chitinases degrade pathogen cell walls to release chitin oligosaccharides (CTOS), which act as elicitors to amplify immunity [23]. We detected no *TRs0x1c000780* expression prior to *P. brassicae* infection. However, at 28 DAI, its expression was induced over 10-fold in the resistant line while remaining silenced in the susceptible line. This induction coincides with the secondary plasmodial development phase of *P. brassicae*. These results suggest that *TRs0x1c000780* represents an inducible candidate defense gene during late-stage infection. This genotype- and stage-specific expression of *TRs0x1c000780* matches the expression profiles of key chitinase genes in related species under clubroot stress. For instance, in *Brassica oleracea*, comparative transcriptome analysis of resistant cabbage cultivars infected by *Plasmodiophora brassicae* revealed that chitinase-encoding genes are key components of the ETI-like response, being dramatically upregulated during the secondary plasmodial development stage to restrict fungal colonization [43]. Furthermore, root RNA-seq profiling of clubroot-resistant and -susceptible Chinese cabbage lines revealed that pathogenesis-related genes, particularly chitinases, are prominently recruited to build a robust physical barrier at late infection stages in resistant hosts [38]. Our localization analysis demonstrated that *TRs0x1c000780* is specifically targeted to the apoplast/extracellular space, as evidenced by its co-localization with the apoplastic marker *AT2G14610.1*-mCherry. This is consistent with studies in other plants showing that Class I chitinases containing an N-terminal signal peptide and chitin-binding domain are targeted to the apoplast to directly contact and degrade invading pathogens [17]. This localized degradation is proposed to release elicitor-active chitin oligosaccharides (CTOS), which are then perceived by cell-surface pattern recognition receptors (PRRs) to amplify immune signaling [8]. Because functional validation through genetic transformation or gene knockouts has not yet been conducted in this study, the transcriptomic induction and apoplastic targeting of *TRs0x1c000780* provide correlative evidence rather than definitive proof of functional causality. Consequently, *TRs0x1c000780* may be a candidate gene for clubroot resistance.

## 4. Materials and Methods

### 4.1. Identification and Physicochemical Properties of Chitinase Genes

To identify chitinase genes in the radish (*Raphanus sativus*) genome, a combined homology-based approach was employed. The reference genome sequence and annotation files of the radish cultivar ‘Xin-li-mei’ (Genome assembly IVFCAAS_Rs00_1.1, GenBank assembly accession: GCA_019703475.1) were acquired from the NCBI GenBank/Raphanus genome database [2]. BLASTP (NCBI; https://blast.ncbi.nlm.nih.gov/Blast.cgi, accessed on 20 July 2026) searches were conducted using 26 *Arabidopsis thaliana* chitinase protein sequences as queries with an E-value threshold of 10^−5^ [17]. Concurrently, Glyco_hydro_18 (PF00184) and Glyco_hydro_19 (PF00182) HMM profiles were acquired from Pfam (http://pfam.xfam.org/, accessed on 20 July 2026) [45]. These profiles were used to screen the *R. sativus* dataset via HMMER 3.0 [46]. Candidate sequences from both methods were merged, and duplicates were removed. We validated the domains of the remaining candidates using the NCBI Conserved Domain Database (https://www.ncbi.nlm.nih.gov/Structure/cdd/cdd.shtml, accessed on 20 July 2026) [47], SMART (http://smart.embl.heidelberg.de/, accessed on 20 July 2026) [48], and Pfam. Proteins containing at least one complete GH18 or GH19 domain were classified as *RsChi* family members. This screen identified 24 high-confidence *RsChi* genes in *R. sativus*. Sequence extraction, filtering, and visualization were conducted using TBtools-II (version 2.066) [49]. Physicochemical properties, including amino acid length, molecular weight, and isoelectric point, were calculated using ExPASy ProtParam (https://web.expasy.org/protparam/, accessed on 20 July 2026) [50].

### 4.2. Chromosomal Location and Gene Nomenclature

Chitinase protein sequences from other Brassicaceae species (*Arabidopsis thaliana*, *Brassica rapa*, and *Brassica juncea*) were acquired from the Phytozome v13 database (https://phytozome-next.jgi.doe.gov/, accessed on 20 July 2026) and NCBI GenBank based on published genome-wide identification studies [12,25]. Chromosomal coordinates of the 24 *RsChi* genes were acquired from the *R. sativus* genome annotation files [2]. To visualize their physical locations, the genes were mapped to chromosomes using TBtools-II [49].

### 4.3. Phylogenetic Analysis

To investigate evolutionary relationships, we conducted a comparative phylogenetic analysis across five Brassicaceae species. We analyzed a total of 172 chitinase sequences, including 24 from *Raphanus sativus*, 64 from *Brassica napus*, 33 from *Brassica rapa*, 25 from *Brassica oleracea*, and 26 from *Arabidopsis thaliana*. The chitinase coding and amino acid sequences for the related Brassicaceae species were acquired from previously published genome-wide identification studies and curated genomic databases (TAIR, BRAD v3.0, and Phytozome v13), specifically utilizing the dataset and identification criteria established by Chen et al. [25] for B. rapa and associated Brassicaceae relatives. All retrieved sequences were re-validated and aligned the presence of intact GH18 (PF00184) or GH19 (PF00182) catalytic domains using HMMER 3.0 and the NCBI Conserved Domain Database before phylogenetic alignment. Multiple sequence alignments conducted using ClustalW (version 1.83) with default parameters [51]. Subsequently, a phylogenetic tree was constructed using MEGA 11 software [52].

### 4.4. Gene Structure, Conserved Motifs, and Domain Analysis

We analyzed *RsChi* exon–intron structures, conserved motifs, and domain architectures to assess structural conservation and divergence. Exon–intron structures of the *RsChi* genes were extracted from the *R. sativus* cv. ‘Xin-li-mei’ annotation files. Conserved protein motifs were identified using MEME (version 5.5.9), with parameters set to detect a maximum of 10 motifs [53]. Conserved domains were annotated using the NCBI Conserved Domain Database [47]. The phylogenetic tree, exon–intron structures, conserved motifs, and domains were visualized using TBtools-II [49].

### 4.5. Prediction of Cis-Acting Elements

To investigate promoter regulation, we analyzed cis-acting elements in the promoter regions of *RsChi* genes. We extracted upstream promoter sequences (2000 bp upstream of the ATG start codon) from the *R. sativus* genome using TBtools-II [2,49]. These sequences were submitted to PlantCARE to predict cis-acting elements [54]. Stress- and phytohormone-responsive cis-acting elements were filtered and visualized using TBtools-II.

### 4.6. Plant Materials, Clubroot Inoculation, and Tissue Sampling

Two long white radish (*Raphanus sativus* L., 2n = 2x = 18) inbred lines, “RB” (highly resistant, resistant to *Plasmodiophora brassicae* race 4) and “SB” (highly susceptible, susceptible), were used as experimental materials in this study. Both inbred lines were developed through successive self-pollination from distinct Korean radish germplasms [2]. Seeds were germinated on moist filter paper at 23 °C for 2 days, transferred to nursery plugs containing a sterile 1:1:1 (*v*/*v*/*v*) mixture of vermiculite, peat moss, and soil, and cultured in a growth chamber at 25 °C/20 °C (day/night) under a 16 h light/8 h dark photoperiod.

The *P. brassicae* isolate utilized was a physiological race 4 strain, according to the Williams pathotype classification system, originally collected from infected radish fields in Xinye County, Nanyang City, Henan Province, China [2]. Resting spores were extracted from mature clubroot galls following cheesecloth filtration and differential centrifugation and resuspended in sterile distilled water to a final density of 3.0 × 10^8^ resting spores/mL [2,43]. Two-week-old seedlings (two true-leaf stage) were inoculated by drenching 5 mL of the resting spore suspension directly around the root zone of each plant. Disease severity was evaluated using a 0 to 4 scale, and the Disease Index (DI) was calculated according to established protocols [4] to confirm contrasting phenotypic responses prior to tissue sampling.

Root tissues were harvested at three biologically distinct infection stages [2]: 0 days after inoculation (DAI, uninoculated baseline control), 7 DAI (corresponding to primary zoospore colonization of root hairs and epidermal cells), and 28 DAI (corresponding to secondary plasmodial development and cortical gall expansion). At each sampling point, roots from five individual plants per genotype were pooled to form one biological replicate, with three independent biological replicates collected (18 samples in total). Samples were immediately frozen in liquid nitrogen and stored at −80 °C prior to total RNA extraction and qRT-PCR analysis.

Two contrasting radish (*Raphanus sativus* L.) inbred lines—a highly resistant line (RB, also referred to as FB) and a highly susceptible line (SB, also referred to as MB)—were utilized in this study. The disease resistance phenotypes of both lines were pre-verified through disease index (DI) scoring following *Plasmodiophora brassicae* inoculation. Resting spores of *P. brassicae* (Williams pathotype 4, the predominant strain in local production) were extracted from infected radish root galls according to established protocols. Spore suspensions were diluted with sterile distilled water to a final concentration of 1.0 × 10^7^ resting spores/mL. Two-week-old radish seedlings grown in a greenhouse (25 °C/20 °C, 16 h light/8 h dark) were inoculated using the root-dipping method for 30 min, followed by transplanting into sterile soil mixtures and drenching with 5 mL of the spore suspension per plant. Root samples were collected at three biologically critical time points: 0 days after inoculation (DAI, uninoculated baseline control), 7 DAI (corresponding to the primary root hair infection stage), and 28 DAI (corresponding to the secondary cortical colonization and gall expansion stage). At each sampling point, roots from five independent plants per line were pooled to form one biological replicate, with three independent biological replicates harvested. Samples were immediately frozen in liquid nitrogen and stored at −80 °C prior to total RNA extraction.

RNA concentration and integrity were assessed using a NanoDrop 2000 spectrophotometer (Thermo Scientific, Waltham, MA, USA) and agarose gel electrophoresis. Subsequently, first-strand cDNA was synthesized from 1 μg of total RNA using the PrimeScript RT Kit with gDNA Eraser (TaKaRa, Beijing, China).

Gene-specific primers for the *RsChi* genes were designed using Primer Premier 5.0. The RsGAPDH gene served as the endogenous control for normalization (Table S1) [55]. Reactions were performed on a Bio-Rad CFX96 Detection System using SYBR Premix Ex Taq II (TaKaRa). Cycling conditions were initial denaturation at 95 °C for 30 s, followed by 40 cycles of 95 °C for 5 s and 60 °C for 30 s. A final melting curve analysis was performed to verify primer specificity. Relative expression levels were calculated using the comparative 2^−ΔΔCT^ method [56]. All assays were performed with three biological and three technical replicates. Statistical significance among multiple genes and sampling time points was determined using two-way ANOVA followed by Benjamini–Hochberg False Discovery Rate (FDR) correction, with adjusted *p* < 0.05 representing statistical significance.

### 4.7. Subcellular Localization Assayss

To establish the subcellular compartmentalization of the promising candidate defense gene, the coding sequence of *TRs0x1c000780* (excluding the stop codon) was amplified from cDNA of the resistant line (RB). The fragment was cloned into the pCNG vector to generate a *TRs0x1c000780*-GFP construct driven by the Cauliflower mosaic virus 35S promoter. For co-localization analysis, a 35S::*AT2G14610.1*-mCherry construct harboring the Arabidopsis marker gene *AT2G14610.1* was utilized (Table S2).

The recombinant plasmids (35S::*TRs0x1c000780*-GFP and 35S::*AT2G14610.1*-mCherry) were transformed into *Agrobacterium tumefaciens* strain GV3101. The positive Agrobacterium strains were cultured, harvested by centrifugation, and resuspended in an infiltration buffer containing 10 mM MES (pH 5.6), 10 mM MgCl_2_, and 200 uM acetosyringone. The bacterial suspensions were adjusted to a final optical density (OD600) of 0.6. For the co-localization assay, Agrobacterium cultures containing *TRs0x1c000780*-GFP and *AT2G14610.1*-mCherry were mixed in a 1:1 volume ratio. Agrobacterium-mediated transient expression was performed by infiltrating the mixture into the abaxial surface of 4-week-old *Nicotiana benthamiana* leaves using a 1 mL needleless syringe. After infiltration, the plants were maintained in a growth chamber at 25 °C under a 16 h/8 h (light/dark) photoperiod. After 48 to 72 h of incubation, the fluorescent signals in the leaf epidermal cells were visualized and imaged using a laser scanning confocal microscope (Zeiss LSM 880, Carl Zeiss, Jena, Germany). EGFP was excited at 488 nm, and emission was collected at 495–530 nm; mCherry was excited at 561 nm, and emission was collected at 580–630 nm.

## Figures and Tables

**Table 1:** Physicochemical properties of *RsChi* genes in *Raphanus sativus*.

Gene ID	Chr.	Number of Amino Acid	Molecular Weight	Theoretical pI	Instability Index	Aliphatic Index	Grand Average of Hydropathicity	E-Value
*TRs0x5c024233*	Chr5	273	29,554.04	4.94	31.52	70.48	−0.097	1.72 × 10^−61^
*TRs0x3c012764*	Chr3	262	28,536.97	4.68	31.89	67.79	−0.125	1.04 × 10^−62^
*TRs0x3c013263*	Chr3	270	28,771.29	8.28	35.2	61.11	−0.187	2.09 × 10^−59^
*TRs0x3c012512*	Chr3	272	28,871.15	8.31	29.59	58.49	−0.226	5.57 × 10^−60^
*TRs0x1c000780*	Chr1	265	29,028.55	8.41	38.79	63.36	−0.265	1.70 × 10^−60^
*TRs0x4c020628*	Chr4	260	28,641.32	5.66	32.28	61.15	−0.13	1.72 × 10^−51^
*TRs0x3c013261*	Chr3	284	30,685.17	5.31	33.62	55.7	−0.363	4.42 × 10^−63^
*TRs0x3c013262*	Chr3	265	28,904.9	8.85	32.34	69.62	−0.051	1.04 × 10^−52^
*TRs0x1c000305*	Chr1	282	30,587.07	5.23	30.57	53.4	−0.345	1.10 × 10^−49^
*TRs0x3c013267*	Chr3	281	30,190.87	9.66	33.91	57.05	−0.204	1.17 × 10^−59^
*TRs0x4c020629*	Chr4	281	30,109.67	9.62	34.73	59.82	−0.228	5.33 × 10^−58^
*TRs0x3c013268*	Chr3	275	29,682.71	8.66	36.84	58.95	−0.152	1.24 × 10^−57^
*TRs0x3c014003*	Chr3	269	29,289.32	9.26	39.87	62.94	−0.268	5.70 × 10^−138^
*TRs0x1c000304*	Chr1	278	29,642.68	9.05	31.99	60.47	−0.166	3.78 × 10^−57^
*TRs0x5c026476*	Chr5	341	37,215.84	8.22	41.54	57.33	−0.379	5.49 × 10^−172^
*TRs0x5c022192*	Chr5	340	37,022.55	5.16	38.7	58.91	−0.289	3.45 × 10^−134^
*TRs0x3c013266*	Chr3	277	31,123.93	8.91	35.93	66.25	−0.176	2.47 × 10^−44^
*TRs0x6c030902*	Chr6	271	30,170.94	6.94	52.12	65.24	−0.297	1.32 × 10^−125^
*TRs0x3c013265*	Chr3	280	31,174.92	9.19	35.8	71.07	−0.263	3.40 × 10^−41^
*TRs0x9c041214*	Chr9	279	31,349.49	8.55	46.5	66.13	−0.328	2.32 × 10^−124^
*TRs0x1c005676*	Chr1	335	36,093.59	5.25	40.81	56.93	−0.325	1.21 × 10^−153^
*TRs0x1c005677*	Chr1	332	35,849.31	5.25	46.04	51.84	−0.351	6.80 × 10^−150^
*TRs0x6c030662*	Chr6	321	35,624.58	6.52	31.74	70.28	−0.185	6.01 × 10^−63^
*TRs0x8c039377*	Chr8	321	35,628.53	6.59	33.67	69.97	−0.228	3.01 × 10^−62^

## Data Availability

The original contributions presented in this study are included in the article/Supplementary Material. Further inquiries can be directed to the corresponding authors.

## Supplementary Materials

The following supporting information can be downloaded at https://www.mdpi.com/article/10.3390/ijms27156765/s1.
